# Suppurative Spondylitis Misdiagnosed as Endplate Inflammation: A Rare Case Report

**DOI:** 10.1002/ccr3.70768

**Published:** 2025-08-07

**Authors:** Cheng Li, Nian‐su Xiao, Tao Xiong, Sen Li, Bao‐yi Ke, Yang Lin

**Affiliations:** ^1^ Guilin People's Hospital China

**Keywords:** infection, mNGS, *Streptococcus agalactiae*, suppurative spondylitis

## Abstract

Spinal infectious diseases are difficult to diagnose and treat; we reported a case of pyogenic spondylitis misdiagnosed as terminal discitis and successfully treated. The application of metagenomic next‐generation sequencing technology holds promise in greatly improving diagnostic efficiency.

## 1 Introduction

Spinal infectious diseases are difficult to diagnose and treat, accounting for 2%–7% of systemic skeletal and muscular system infections [1]. It has been reported in the literature that the incidence rate among spinal infection patients is approximately 1/250,000–1/100,000, with the mortality rate ranging from 2% – 4% [2]. With the increasing number of spinal surgical procedures, a rise in immunodeficiency patients, and advancements in diagnostic and treatment techniques, the incidence rate of spinal infectious diseases is increasing year by year [3], with a simultaneous increase in the rates of missed diagnosis and misdiagnosis of spinal infectious diseases. First, the clinical and imaging characteristics of spinal infections are relatively similar to some spinal tumors, seronegative spondylitis with fractures, endplate inflammation, and spinal trauma, thereby making it difficult to accurately differentiate [4]. Second, the clinical characteristics, signs, and imaging features of the pathogenic microorganisms responsible for spinal infections, including pyogenic bacteria, *Brucella*, *Mycobacterium tuberculosis* , and fungi, often lack specificity, leading to confusion in establishing diagnosis [5]. This study presents a case of pyogenic spondylitis infection caused by *Streptococcus agalactiae* . Due to the rarity of spinal infections caused by *Streptococcus agalactiae* , this study reviews the clinical and imaging findings, diagnosis, treatment strategies, and prognosis of this disease to improve clinicians' understanding of this disease.

## 2 Case History/Examination

**FIGURE 1:**
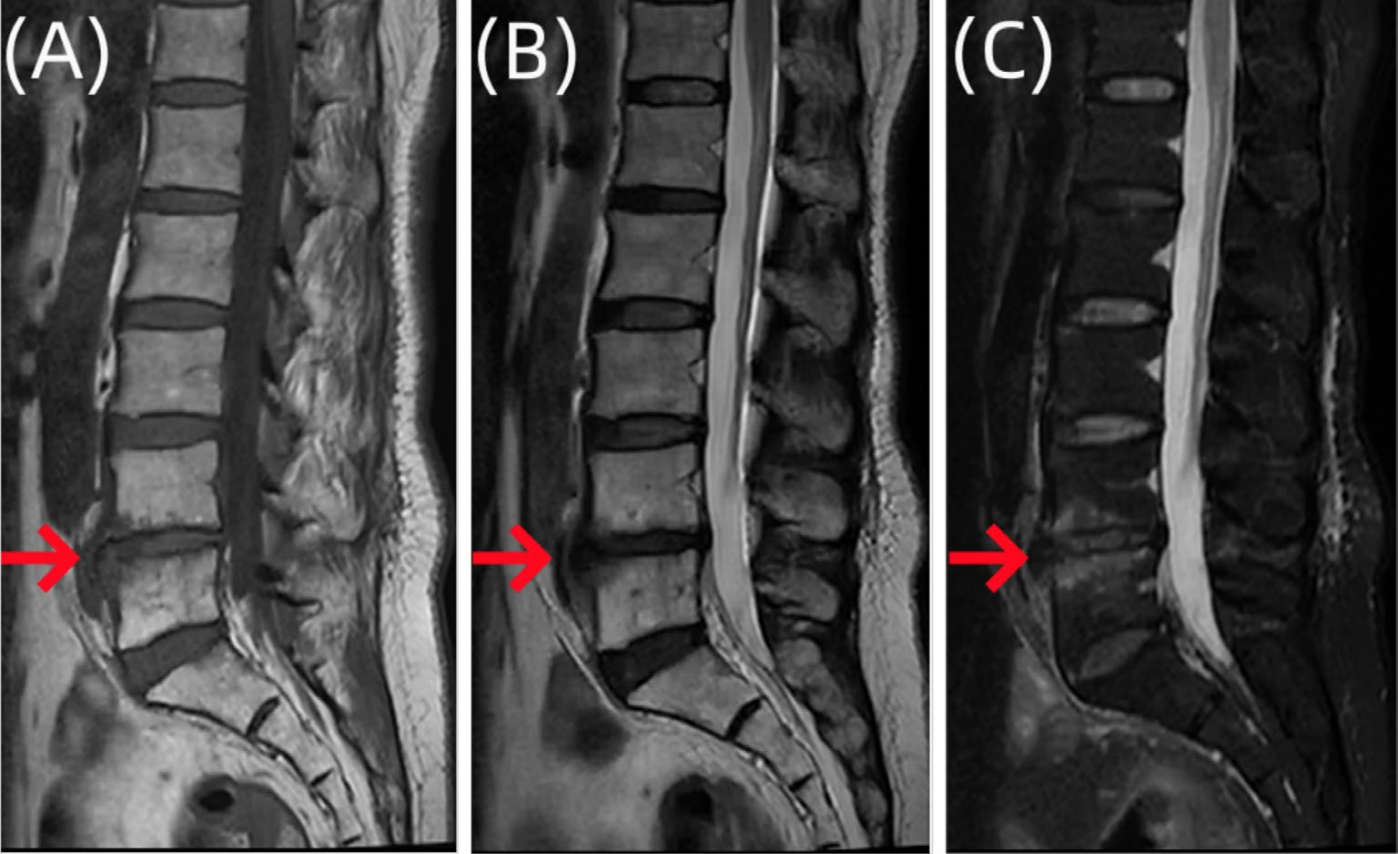
Sagittal images of lumbar vertebral MR examination of the patient. Imaging examination showed that the edges of L4 and L5 vertebral bodies showed strips of abnormal signal images, (A) T1W1 showed slightly low signal, (B) T2W1 showed slightly high signal, (C) Fat suppression image showed slightly high signal, L4/5 intervertebral space narrowed, considered as L4 and L5 vertebral endplate inflammation.

The patient is a 64‐year‐old woman who visited the outpatient clinic of our hospital for the first time in April 2023. She presented with a complaint of lower back pain with a Visual Analog Scale (VAS) score of 4 points, with no obvious radiating pain and numbness in the lower limbs, and both lower limbs exhibited normal sensation and muscle strength. A complete lumbar spine magnetic resonance imaging (MRI) was performed at the outpatient clinic (Figure 1). Based on the patient's symptoms, signs, and imaging examinations, the outpatient doctor considered that the patient's lower back pain symptoms were caused by lumbar 4/5 segment endplate inflammation in conjunction with lumbar disc herniation. The patient was recommended bed rest and prescribed some nonsteroidal anti‐inflammatory and analgesic drugs.

**FIGURE 2:**
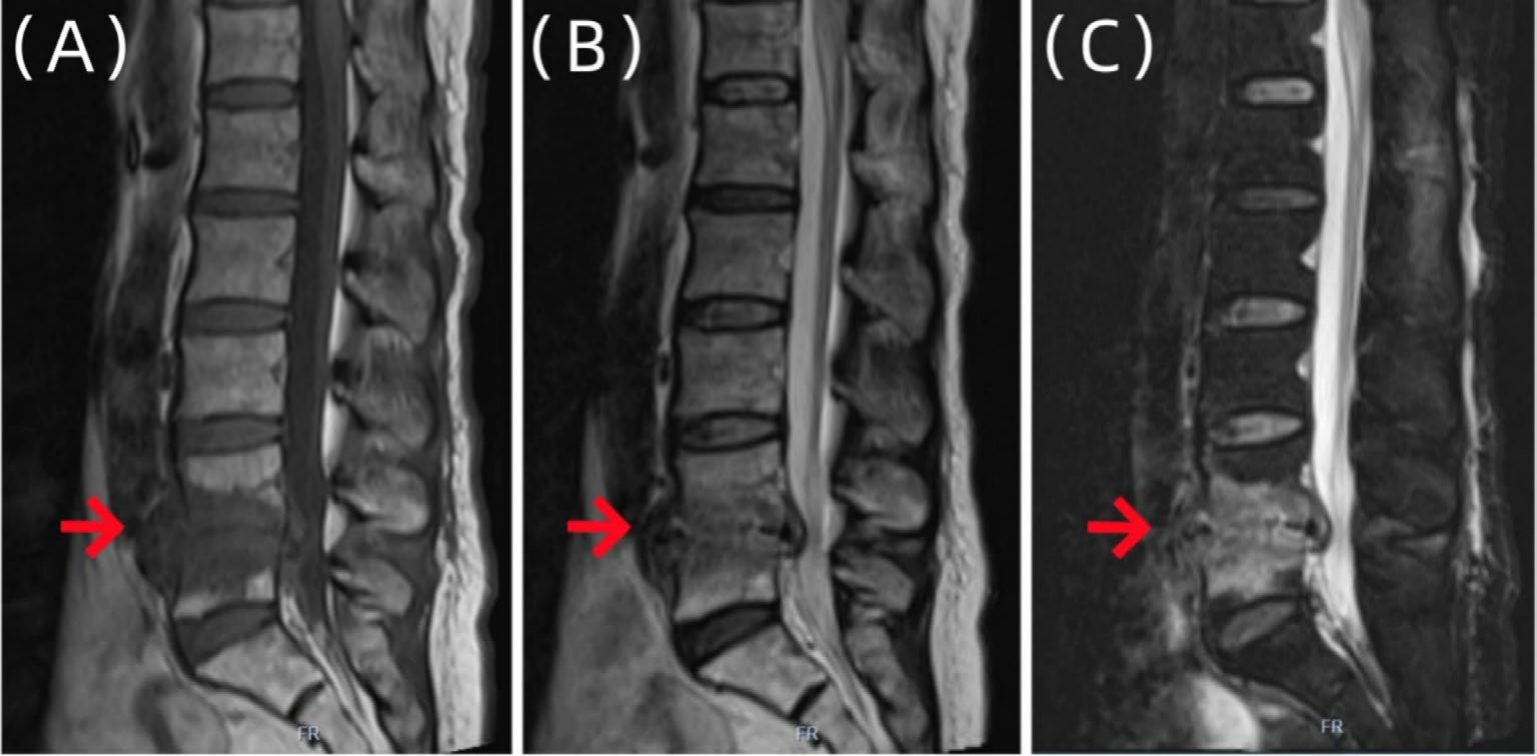
The sagittal image of the patient's lumbar spine MR examination. The imaging examination showed that the signal of L4 and L5 vertebral bodies was uneven. (A) The signal of T1W1 was low. (B) The lipid pressure of T2W1 was high. (C) And the intervertebral space of L4/5 was narrowed. Spinal infection was considered.

**FIGURE 3:**
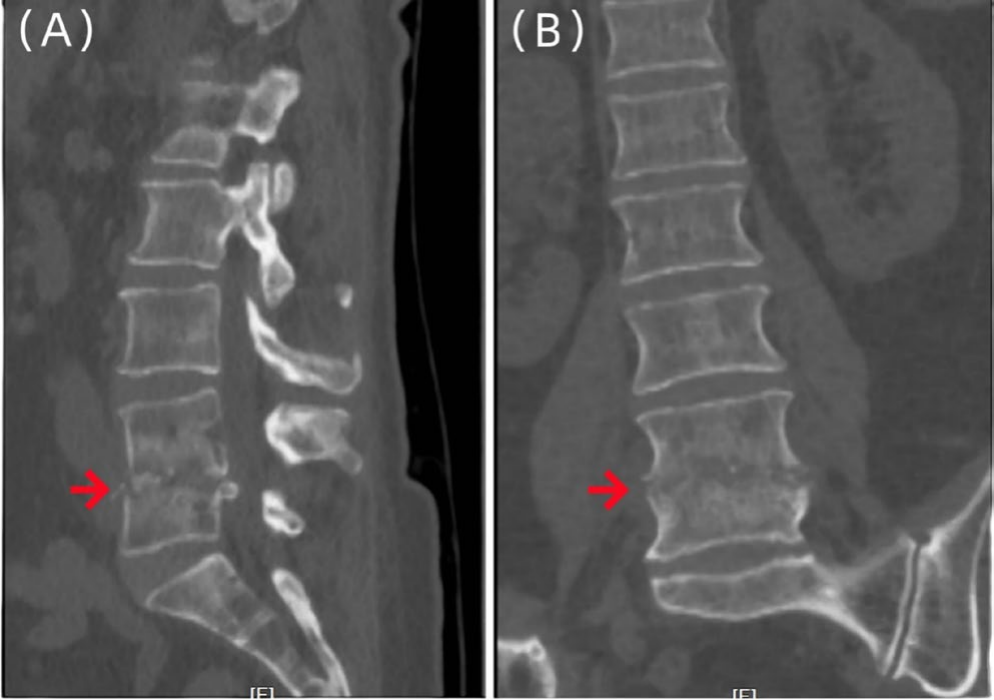
Three‐dimensional CT images of the lumbar vertebrae of the patient. Imaging examinations showed irregular bone destruction in the lower part of the L4 vertebral body and the upper part of the L5 vertebral body, the destruction and narrowing of the L4/5 intervertebral disc, and the spindle‐shaped soft tissue swelling in the paravertebral body (A–B).

However, the patient came back to the outpatient clinic of our hospital in May 2023, reporting exacerbated lower back pain and progressive radiating pain in both lower limbs (mainly in the area innervated by the L5 nerve root), with a VAS score of 8 points. Lumbar spine MR (Figure 2) and lumbar spine 3D computed tomography (CT) (Figure 3) were performed at the outpatient clinic, and an initial diagnosis of spinal infection was reached. The patient was hospitalized for further examination and treatment.

Following hospitalization, the patient's physical examination showed normal vital signs, a painful face, and forced hip flexion and knee flexion in a passive sitting position. There was limited flexion, extension, and rotation of the lumbar vertebrae, with tenderness and percussion pain noted in the spinous process and paravertebral process of the lumbosacral region, accompanied by radiating pain in both buttocks, posterolateral thigh, lateral calf, and back of the foot. The findings revealed a positive straight leg elevation test of both lower limbs (approximately 40° positive) and a positive reinforcement test, normal muscle strength and tension in the lower limbs, and negative tendon reflexes.

Through comprehensive medical history collection, physical examination, and laboratory evaluation, we confirmed the absence of peripheral joint arthritis, dactylitis (sausage digits), psoriatic skin lesions, heel pain, or a history of uveitis. The patient had no family history of spondyloarthritis and denied prior episodes of septic arthritis or intra‐articular infections. Before symptom onset, there was no history of trauma, invasive procedures (e.g., joint aspiration), or exposure to identifiable infectious foci. Further evaluation ruled out underlying conditions such as diabetes mellitus, chronic kidney disease, or malignancy, with no documented prolonged use of glucocorticoids, biologics, or other immunosuppressive therapies. The patient maintained good general health prior to this episode, with no identifiable risk factors for compromised immune function.

History: The patient had undergone a total hysterectomy due to a prior diagnosis of a cervical malignant tumor and had a history of vesicovaginal fistula.

Laboratory examination showed that white blood cell count and procalcitonin levels were normal; C‐reactive protein (CRP) and interleukin levels and erythrocyte sedimentation rate (ESR) were markedly elevated; specific IgG and IgM antibody of *Mycobacterium tuberculosis* were negative; pure protein derivative test of tuberculin was negative; T cell spot test of *Mycobacterium tuberculosis* was negative; tumor markers were negative; and blood culture was negative.

## 3 Methods

**FIGURE 4:**
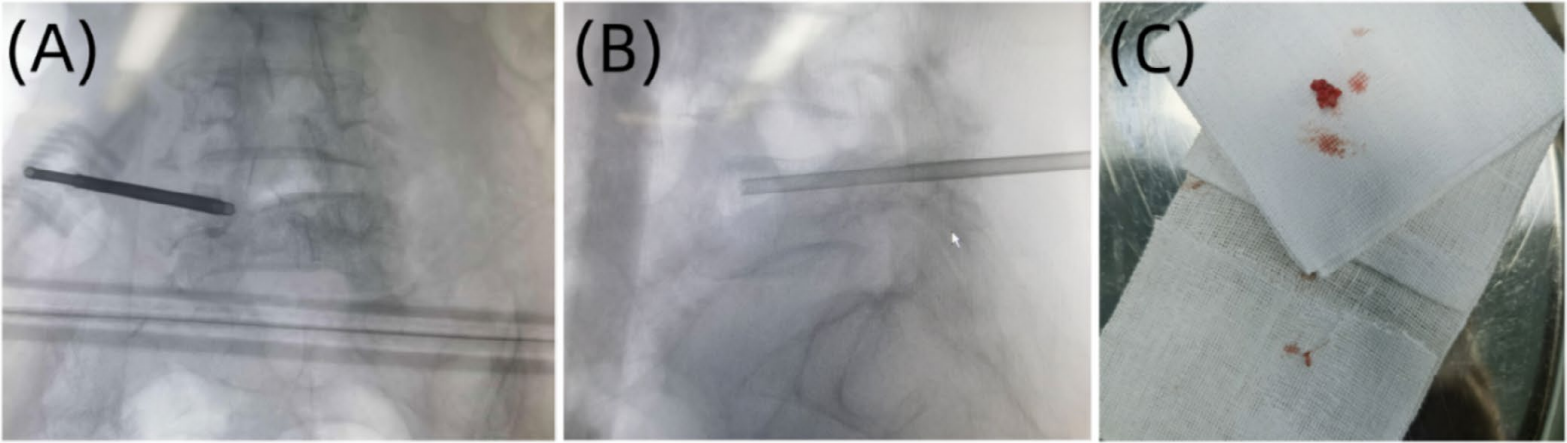
The patient underwent a C‐arm fluoroscopy‐guided puncture of the lesion tissue of the lumbar 5 vertebral body. (A) X‐ray anteroposterior fluoroscopic images of the patient's lesion tissue taken during the operation; (B) X‐ray lateral fluoroscopic images of the patient's lesion tissue taken during the operation; (C) The lesion tissue obtained at the end.

The patient was advised to undergo a percutaneous biopsy of the vertebral body as shown in Figure 4. During the procedure, tissue samples from the lesion tissue of the lumbar 5 vertebral body were taken and sent to culture, pathological examination, and second‐generation metagenomic sequencing, respectively. The culture results were negative, and no tumor lesions were found in the pathological examination. However, the second‐generation metagenomic sequencing results revealed the presence of *Streptococcus agalactiae* (number of detected sequences: 830 and a confidence level of 99%).

## 4 Conclusion and Results

**FIGURE 5:**
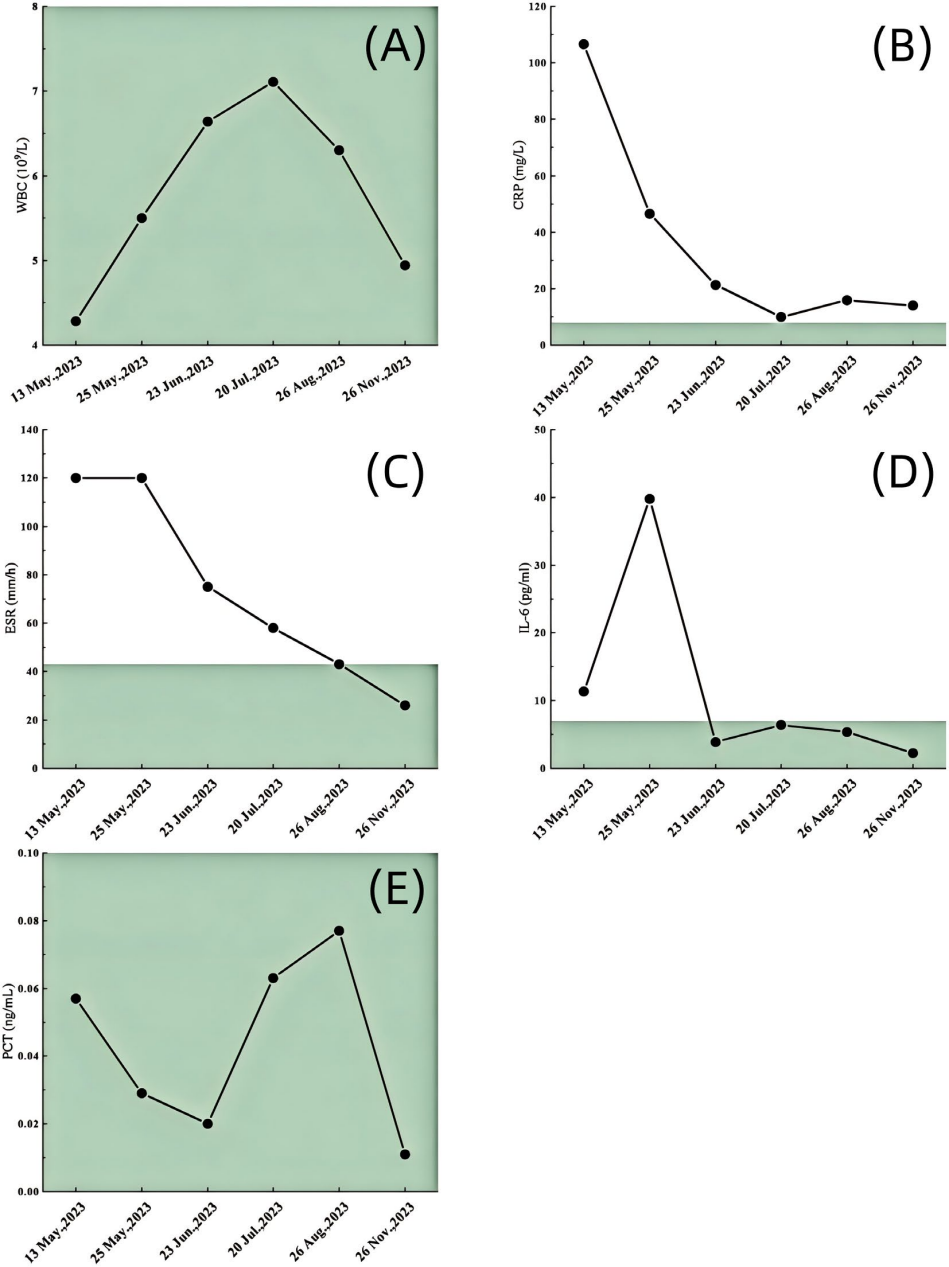
Fluctuations in various indicators related to inflammation and antibiotic use. Reference value (A–E): WBC 3.5 × 10^9^/L – 9.5 × 10^9^/L; CRP 0–8 mg/L; ESR 0–43 mm/h; PCT 0–0.10 ng/mL. IL‐6 0–7 pg/mL; WBC: White blood cell; CRP: C‐reactive protein; ESR: Erythrocyte sedimentation rate; PCT: Procalcitonin; IL‐6: Interleukin‐6.

**FIGURE 6:**
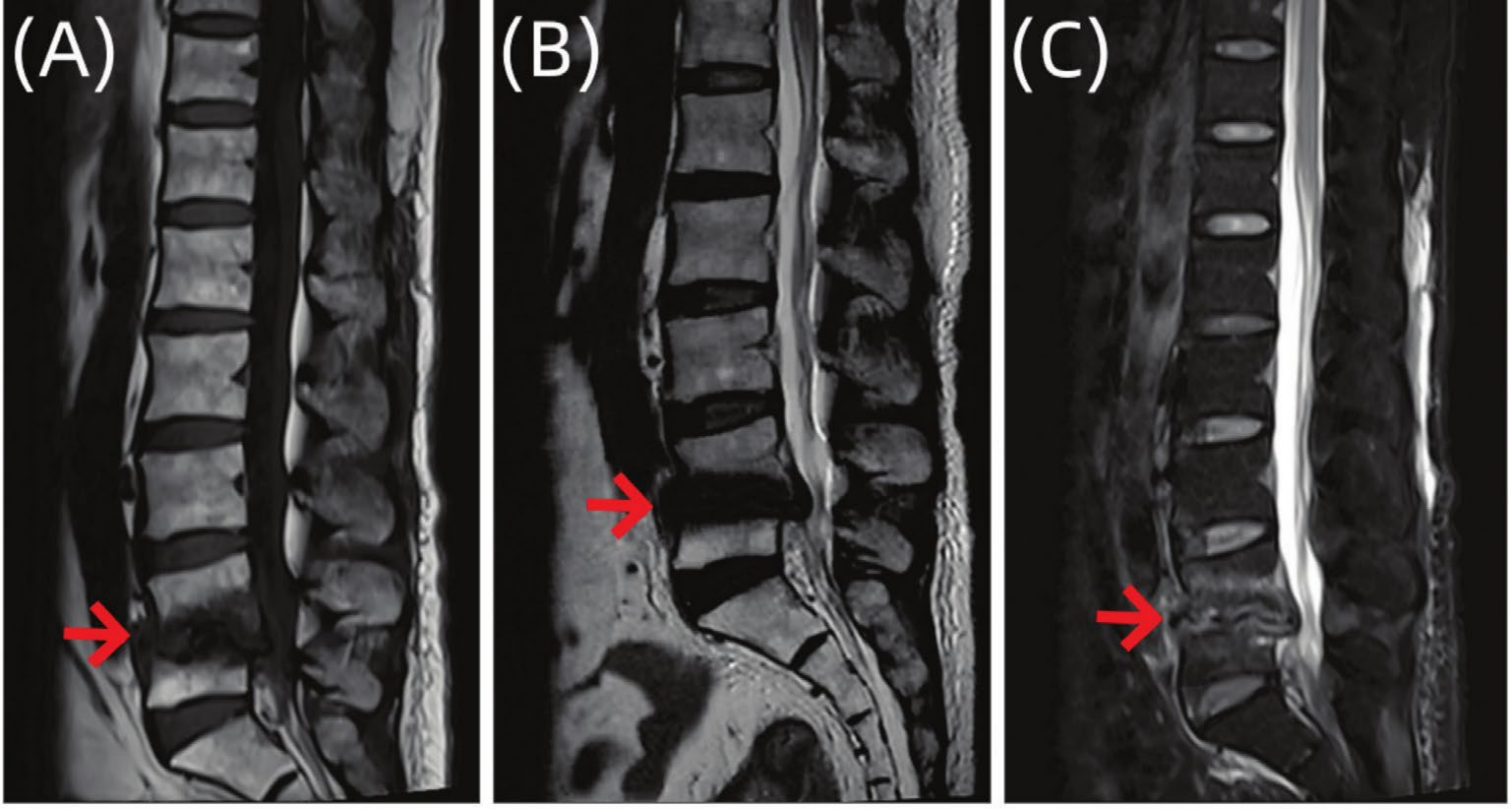
Sagittal MR images of the lumbar vertebrae of the patient. Imaging examination showed that the morphology of L4 and L5 vertebral bodies was irregular, and the edges of L4 and L5 vertebral bodies showed strips of abnormal signals, which were significantly reduced compared with (Figure 2) A–C.

**FIGURE 7:**
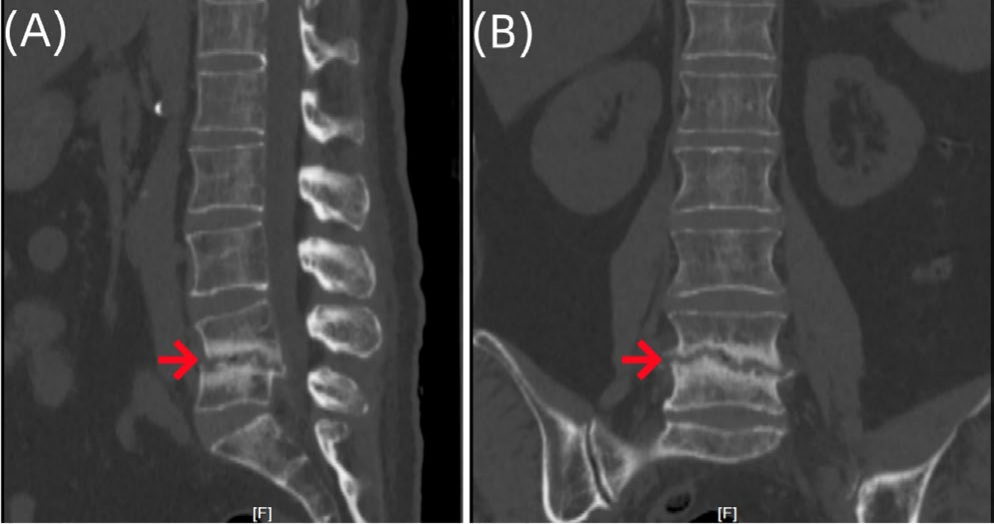
Three‐dimensional CT images of the lumbar vertebrae of the patient. Imaging examinations showed that compared with (Figure 3), L4 and L5 vertebral bodies were destroyed, and local bone mineral density was higher than before (A–B).

**FIGURE 8:**
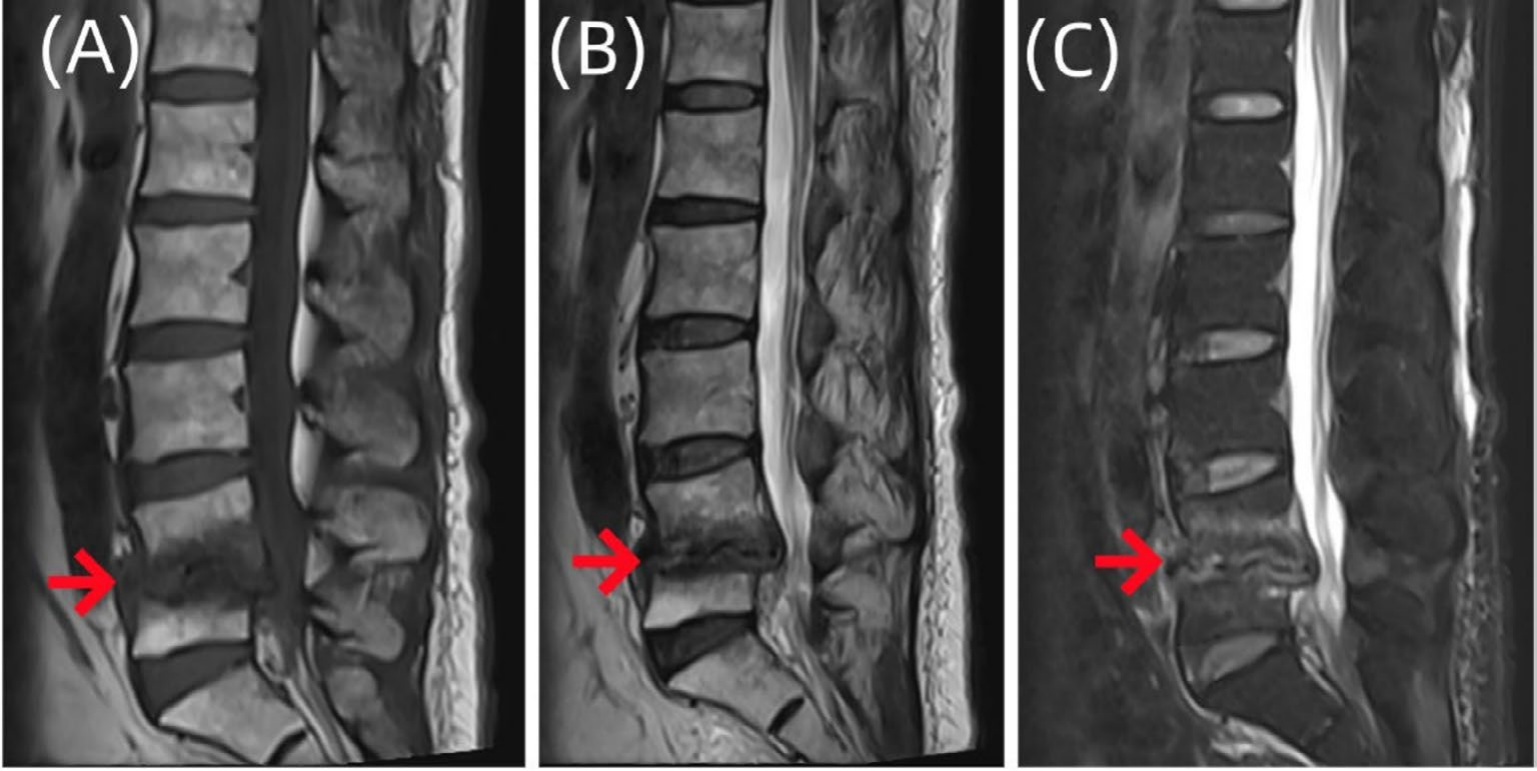
Sagittal MR images of the lumbar vertebrae of the patient. Imaging examination showed that the morphology of L4 and L5 vertebral bodies was irregular, and the edges of L4 and L5 vertebral bodies showed strips of abnormal signal shadows (A–C), which were slightly less than those in Figure 5A–C.

**FIGURE 9:**
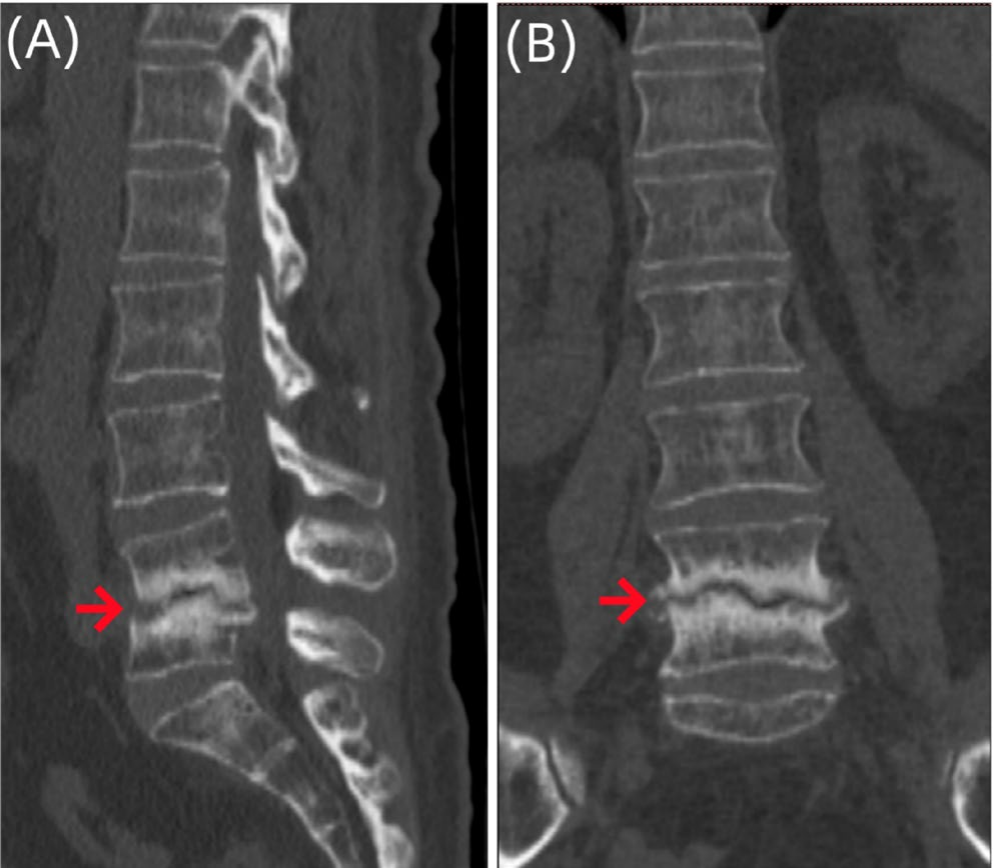
Three‐dimensional CT images of the lumbar vertebrae of the patient. Imaging examination showed that compared with Figure 6, there was no increase in bone destruction in L4 and L5 vertebrae, and the local bone mineral density was higher than before (A–B).

Considering the patient's medical history, symptoms, and signs, the patient was diagnosed with suppurative spondylitis caused by *Streptococcus agalactiae* infection. The patient was discharged after an intravenous infusion of cefazolin sodium (1 g, Q8h) for 1 week and advised to continue taking oral cefadroxil chewable tablets for 3 months after discharge (1 g, Bid). After completing a 1‐week course of antibiotics, the patient experienced substantial improvement in the lower back pain and radiating pain in both lower limbs. At the same time, the patient was treated in the first week, 1, 2, 3, and 6 months after antibiotic application. Inflammatory indicators were reviewed (as shown in Figure 5). In the 3rd month (Figures 6, 7) and 6th month (Figures 8, 9), MRI and CT scans were performed respectively. The combined analysis of inflammatory indices and imaging findings revealed a gradual decrease in the infection indices of the patient, a substantial reduction in the edema signal of the lumbar 4/5 segment, formation of new sclerotic bone, and stabilization of the lesion segment of the spine. Conservative treatment proved effective. At the 1‐year follow‐up, the patient reported complete relief from the lower back pain and radial pain in both lower limbs and returned to normal daily activities.

## 5 Discussion

The clinical presentation of suppurative spondylitis lacks specificity, and the early symptoms are hidden, which can easily go unnoticed by patients and physicians. Pain at the lesion site is the most common symptom in the early stage of suppurative spondylitis [6], and we should be highly vigilant against suppurative spondylitis if the pain intensifies at night. Fever is also an important symptom of suppurative spondylitis, accounting for approximately 48% of cases. The impaired neurological function represents another hallmark symptom, characterized by numbness and weakness of limbs, sensory disturbance, fecal incontinence, and paralysis, accounting for approximately 32% of cases. These symptoms often arise from direct or indirect compression of nerves by abscesses [7]. Pyogenic spondylitis accounts for approximately 4% of all cases of osteomyelitis, most of which occur in older people aged 50–70 years. This condition is more prevalent in men than women, with a male‐to‐female ratio of 1.5–2: 1. *Staphylococcus aureus* is the most common pathogen, followed by thoracic and cervical vertebrae. Other causative agents include *Staphylococcus albus* and *Pseudomonas aeruginosa* , among others. *Streptococcus* is a gram‐positive, catalase‐negative, coagulase‐negative cocci, occurring in pairs or chains [8]. *Streptococcus agalactiae* is an important cause of puerperal sepsis and neonatal meningitis [9]. It often parasitizes the reproductive tract of pregnant women, can cause infantile infection, and can also cause postpartum infection, bacteremia, meningitis, skin and soft tissue infection, and osteomyelitis [10, 11, 12]. Our patients have a history. This may be one of the predisposing factors of *S. agalactiae* spinal infection in this patient. Through a thorough literature search, it was found that there is a paucity of reported cases of suppurative spinal infections caused by *S. agalactiae* . Therefore, this study aims to present a unique case of suppurative spondylitis resulting from *S. agalactiae* infection, initially misdiagnosed as endplate inflammation, but finally confirmed through mNGS detection. A case of suppurative spondylitis caused by *S. agalactiae* infection was reported.

Due to the nonspecific nature of the clinical manifestations and atypical early symptoms of suppurative spondylitis, it usually takes 30 to 90 days from the onset of symptoms to reach a definitive diagnosis, and some patients even need diagnostic treatment to confirm the diagnosis [13]. MRI is the most sensitive imaging examination method for the early detection of spinal infections. However, MRI findings may be nonspecific, especially in cases with previous spinal surgery, where abscesses and hematomas can exhibit similar imaging features [14]. During the first visit, the patient presented with complaints of low back pain, with no fever and other signs of infection. Clinical symptoms were not specific to lumbar degeneration, and the findings from the lumbar spine MRI revealed patchy abnormal signals at the edges of L4 and L5 vertebral bodies. These signals appeared slightly low on T1WI and slightly high on T2WI and demonstrated slightly high signals on fat suppression images. L4/5 intervertebral space narrowing was difficult, which was roughly the same as that of general disc degeneration. It was challenging to distinguish these symptoms, signs, and imaging findings between specific and nonspecific lumbar spine infections [15]. Therefore, this patient was initially misdiagnosed as endplate inflammation. Tissue samples from the affected vertebral body tissue were obtained under the guidance of C‐arm fluoroscopy, and the bacterial culture yielded negative results. The results of the mNGS revealed *Streptococcus agalactiae* infection. The research team recommends [16] that routine mNGS detection of diseased tissue samples in patients with suspected spinal infections can effectively increase the detection rate in these patients while simultaneously reducing the risk of misdiagnosis and missed diagnosis. Numerous studies [17, 18] have demonstrated that mNGS exhibits remarkable pathogen detection capability in patients with spinal infections. Notably, its diagnostic sensitivity is significantly superior to conventional methods, particularly in cases complicated by opportunistic fungal or viral co‐infections.

At present, there exists no standardized guideline for the treatment of suppurative spondylitis caused by *Streptococcus agalactiae* infection, at home and abroad. Clinically, most cases of intervertebral space infections can achieve good clinical results through conservative treatment. Conservative treatment typically involves the use of sensitive antibiotics and external fixation. The duration of antibiotic therapy for spinal infections varies, usually more than 6 weeks [19, 20], but there are reports [21] that recommend intravenous use of sensitive antibiotics for 2–4 weeks, followed by oral antibiotics for 4–6 weeks. In addition, the route of antibiotic administration remains controversial, but currently, it is generally recommended to use antibiotics for 4–12 weeks for patients with spinal infections [22]. Criteria for discontinuing antibiotic therapy in spinal infections include [23]: (1) substantial relief or disappearance of symptoms; (2) stable leukocyte count, ESR, and CRP levels for 3 consecutive weeks; and (3) resolution of inflammation and edema signals in the vertebral body and intervertebral space, absence of abscess in the spinal canal, and stable spinal sequence. Surgical treatment is required for patients with poor response to conservative treatment [24, 25]. The purpose of surgical treatment is to completely clear the lesion, stabilize the spinal structure, and maintain the stability of the lesion segment of the spinal column and prevent deformities through effective internal fixation. In this case, after 1 week of effective antibiotic therapy, lower back pain and radiating pain in both lower limbs were substantially relieved. After getting out of bed, reliable external fixation braces were used. The inflammatory indicators tended to be normal after 3 months of effective antibiotic use. Abnormal signal absorption on imaging examination, new sclerotic bone formation, so conservative treatment is effective.

In summary, with the aging population, the incidence of suppurative spondylitis is on the rise. Although the advancements in treatment strategies have improved cure rates, early diagnosis remains a thorny problem at present, a crucial factor affecting the prognosis of the disease. C‐arm or O‐arm‐guided puncture biopsy and bacterial culture serve as effective methods for definite diagnosis. Moreover, the application of mNGS technology holds promise in greatly improving the diagnosis efficiency.

## Author Contributions

**Cheng Li:** conceptualization, data curation, writing – review and editing. **Nian‐su Xiao:** conceptualization, writing – review and editing. **Tao Xiong:** data curation. **Sen Li:** funding acquisition, visualization. **Bao‐yi Ke:** data curation, software. **Yang Lin:** funding acquisition, writing – original draft, writing – review and editing.

## Ethics Statement

The study was approved by the licensing ethical committee of Guilin People's Hospital. Written informed consent was obtained from the patient for publication of this case report and accompanying images, complying with the requirements mentioned in Wiley's CCR Consent Form. Informed written consent was obtained from the patient for publication of this case report in a scientific journal.

## Consent

The authors have nothing to report.

## Conflicts of Interest

The authors declare no conflicts of interest.

## Data Availability

The data that support the findings of this study are available from the corresponding author upon reasonable request.
